# Evaluation of Collagen Fibers, MMP2, MMP9, 8-OHdG and Apoptosis in the Aorta of Ovariectomized LDL Knockout Mice Submitted to Aerobic Exercise

**DOI:** 10.5935/abc.20180263

**Published:** 2019-02

**Authors:** Laura Beatriz M. Maifrino, Nathalia E. A. de Lima, Mara R. Marques, Clever G. Cardoso, Lidiane B. de Souza, Tabata de Carvalho Tomé, Hananiah Tardivo Quintana, Flavia de Oliveira, Beatriz da Costa Aguiar Alves Reis, Fernando Luiz Affonso Fonseca

**Affiliations:** 1 Universidade São Judas Tadeu, São Paulo, SP - Brazil; 2 Universidade Federal de Goiás, Goiânia, GO - Brazil; 3 Departamento de Biociências da Universidade Federal de São Paulo, São Paulo, SP - Brazil; 4 Faculdade de Medicina do ABC, Santo André, SP - Brazil

**Keywords:** Rats, Cardiovascuar Diseases, Menopause, Fibrillar Collagens/analysis, Ovariectomy, Exercise, Cholesterol, LDL

## Abstract

**Background:**

In menopause, there is greater cellular exposure to oxidative stress, related to the decreased antioxidative effects of estrogen. These metabolic changes favor the progression of cardiovascular diseases, such as atherosclerosis. Abnormal function of the aorta - the most important artery - is associated with many cardiovascular diseases. Collagen, especially types I and III, is one of the most important aortic wall components and it can be affected by many factors, including menopause. The 8-OHdG is one of the main markers of DNA oxidative damage induced by reactive oxygen species (ROS).

**Objective:**

We aimed to investigate effects of moderate aerobic training on the ascending aorta of LDL-knockout (LDL-KO) and ovariectomized female mice.

**Methods:**

A total of 15 C57BL/6 mice and 15 LDL-KO mice were divided into experimental groups. The thickness and volume density of types I and III collagen fibers were performed by morphoquantitative analysis, whereas the MMP-2 and MMP-9 and 8-OHdG were detected by immunohistochemistry and apoptosis was detected by the TUNEL assay. The significance level for all tests was p < 0.05.

**Results:**

Exercise causes an increase in the thickness of the aorta in LDL-KO groups, particularly accentuated in the ovariectomized groups. The type I collagen fibers showed an increase in volume density influenced by training in both Control groups and in the LDL-KO group. Type III collagen density decreased in both groups. The MMP-2 showed moderade immunostaining in the tunica media in LDL-KO groups, which did not occur in the control groups and the MMP-9 stained irregularly in all tissues. The marker 8-OhdG was stronger in the exercise training groups. Additionally, the ovariectomy, the exercise training and the LDL-KO treatments increased apoptosis.

**Conclusion:**

These results suggest that moderate-intensity aerobic exercise in ovariectomized mice associated to an increase in LDL rate possibly increases oxidative stress and apoptosis induction.

## Introduction

Menopause is a period during which women suffer changes in metabolic profile due to decreased production of hormones such as estrogen.^1-3^ These metabolic changes favor the progression of cardiovascular diseases, such as atherosclerosis.^4^ Abnormal function of the aorta - the most important artery - is associated with many cardiovascular diseases. Collagen, especially types I and III, is one of the most important aortic wall components and it can be affected by many factors, including menopause.^5^

Physical exercises are recommended for preventing cardiovascular diseases during menopause.^6,7^ However, moderate-to-high intensity physical activity causes increased oxidative stress in cells and tissues, raising the risk of cardiovascular disease.^8-10^ The adaptation of the body to oxidative stress may be impaired in individuals with low levels of estrogen, which binds to specific cellular receptors and accelerate the production of various antioxidants by cells.

Little is known about the effects of physical activity on the development of atherosclerosis and metabolic changes that are characteristic of menopause. Relevant data for the elucidation of these effects have been obtained with the use of markers such as 8-hydroxydeoxyguanosine (8-OHdG), metalloproteinases (MMPs), apoptosis detection and quantification of collagen types III and I. 8-OHdG is one of the main markers of DNA oxidative damage induced by reactive oxygen species (ROS).^11,12^ MMPs play key roles in the function of various tissues during growth, development and aging of the organism.^13-17^ The excessive or unbalanced MMP activity is associated with the pathogenesis of many diseases.^18,19^ among them cardiovascular diseases, such as atherosclerosis.^20^

The detection of apoptosis in tissues is a marker related to mitochondrial injury, reactive oxygen species production, and oxidative stress. In apoptosis, DNA breakage results in several fragments with free 3’-OH ends. The identification of cells undergoing the process of apoptosis consists in detecting enzymatically the free 3’-OH ends with the addition of nucleotides modified by the TdT enzyme (terminal deoxynucleotidyl transferase).

Thus, we aimed to verify the effects of moderate aerobic training on the ascending aorta of, low-density lipoprotein receptor LDL knockout and ovariectomized female mice.

## Methods

### Animals and group formation

The experiments were performed in 15 female mice C57BL/6 and 15 of low-density lipoprotein receptor knockout female mice (LDL-KO) weighing 20-25g, from the Animal House of the São Judas Tadeu University, São Paulo, Brazil. The mice received the standard laboratory chow and water *ad libitum*. The animals were placed in cages in a room with controlled temperature (22°C) and a 12-h light-dark cycle. All surgical procedures and protocols were approved by the Experimental Animal Use Committee of Universidade São Judas Tadeu (058/2007). After a simple randomization, the mice were divided into six groups (n = 5): sedentary control (S-C), ovariectomized sedentary control (OS-C), ovariectomized trained control (OT-C), sedentary LDL KO (S-LDL KO), ovariectomized sedentary LDL KO (OS-LDL KO) and ovariectomized trained LDL KO (OT-LDL KO). The animals were separated physically and randomly between the groups / boxes.

The sample size definition was performed according to previous data from other authors,^21-23^ which were based on the instructions of CONCEA (Conselho Nacional de Controle de Experimentação Animal) Normative Instruction N°. 27 and determined by the formula n = (2α/2δ) 2/E^24^ was used, where n stands for sample size; (2α)^2^ stands for a critical value that corresponds to the desired degree of confidence, δ stands for the population standard deviation and E stands for the margin of error (difference between the sample mean and the mean of the true population).

### Ovariectomy

At nine months of age, the animals were anesthetized (ketamine 120 mg/kg + xylazine 20 mg/kg), and a small abdominal incision was performed where the ovaries and oviducts were found, sectioned and removed. Then, the skin and muscle wall were sutured.^25,26^ The efficacy of the ovariectomy was determined by observation of vaginal secretions during four consecutive days.

### Training protocol

Seven days after the ovariectomy, all animals were adapted on the treadmill for ten minutes during three days before initiating the training. The maximal exercise test was performed in all groups at the beginning and at the ending of the training program, providing the basis for the prescription of physical training, and with the purpose to evaluate the physical capacity of the trained animals.

The trained groups were submitted to a moderate physical training protocol on a treadmill, with progressive speed and load (1 hour a day/5 days a week at 50-60% of maximum effort speed) during 4 weeks, as previously described.^27^

### Histological procedures

At the end of the experiment, the animals were weighed and subsequently euthanized by decapitation. Thoracotomy was performed by cutting the ascending aorta at the heart base. The aorta samples were washed (PBS - phosphate buffered saline 0.1M, pH 7.4) and fixed in 10% formaldehyde for 24 hours. Then, they were dehydrated, cleared and embedded in paraffin. The aorta was sectioned transversely (5 µm thick), and the samples were stained in H&E for histomorphometric analysis.^7^ In order to analyze the effects of aerobic training on the whole aorta, the tunica intima was not separated from the tunica media. Picrosirius staining was used for classification of collagen fibers I and III. The images were captured at 4 points, at 0º, 90º, 180º and 270º, with x10 magnification to measure the aorta thickness, and x40 for other evaluations, and transferred to an image analysis program (Axion Visio Software, Zeiss^®^). To analyze the volume density of types I and III collagen fibers, the images were captured by light microscope with polarized light, analyzed using a test system of 252 points, and the values were expressed as percentages.

### Immunohistochemical analysis: 8-OHdG, MMP-2 and MMP-9

Five 4-µm cross-sections, mounted on previously silanized slides, were used to show the expression of 8-OHdG, MMP-2 and MMP-9. The slides were then deparaffinized, cleared, hydrated and washed in running water. Then, endogenous peroxidase activity was blocked with 0.3% hydrogen peroxide, protein blocking was performed with 0.3% skim milk diluted in PBS and the slides were incubated overnight with anti-8-OHdG (SC66036 Santa Cruz® Biotechnology, CA, USA) primary antibody, titrated 1:100, and MMP2/72KDa and MMP9/KDa (SC-10436 Santa Cruz® Biotechnology, CA, USA; SC-6840 Santa Cruz^®^ Biotechnology, CA, USA), titrated 1:150 in PBS-BSA 0.1%. All slides were then placed in a humid chamber at 4°C overnight. The material was washed with PBS buffer and incubated with biotinylated secondary antibody. For revelation, the 3-3´diaminobenzidine chromogenic substrate was used at a ratio of 0.06 g per 100 mL of PBS, and 1 mL of 20-volume H_2_O_2_ for five minutes at 37°C and counter-stained with Mayer’s hematoxylin for 3 minutes. Finally, the slides were mounted with cover slips and entellan® for analysis under light microscopy.

### Investigation of the apoptotic cell death by TUNEL immunocytochemistry

TUNEL staining was performed using the ApopTag Peroxidase In Situ Apoptosis Detection Kit (Millipore^®^, Germany) according to the manufacturer’s instructions.

### Quantification of apoptotic cells

For the quantification of immunostained cells for apoptosis, 30 images of the intima-media layer were captured (10 images/animal; n = 3 animals/group; with x10 magnification to measure the aorta thickness, and x40 for other evaluations, and transferred to an image analysis program (Axion Visio Software, Zeiss^®^) for each experimental group. For each image, the total number of immunostained cells was obtained as a relative frequency (%) in relation to the total number of cells. The light microscope coupled to a digital camera (Zeiss, Germany) was used to obtain the images, and the photomicrographs were scanned with the AxioVision software (Zeiss®, Germany).

### Statistical analysis

The results were presented as mean and standard deviation. Analysis of variance (ANOVA) and Tukey’s post-hoc tests were properly applied in data analysis. The significance level for all tests was p < 0.05. The data were evaluated using the software Stata 7.0. All continuous variables were normally distributed (Shapiro-Wilk test). To evaluate the normality of the data, the Shapiro-Wilk calculation was used, which found that the data were allocated within the Gaussian curve. Considering a level of significance of 5%, the test assumed the normality hypothesis for the variable with normal distribution.

## Results

### Histopathological and histomorphometric analysis

**Figure 1:**
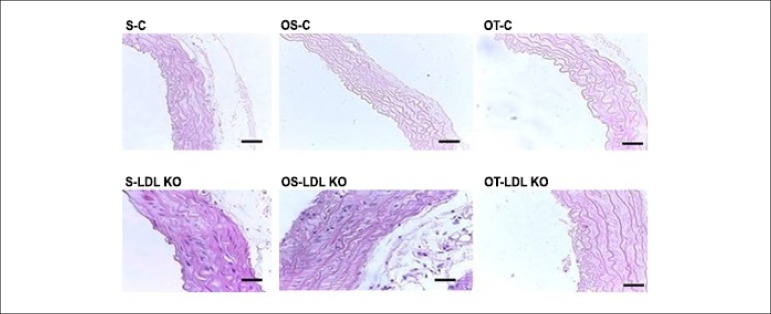
Mouse aorta cross-sections showing the arrangement of elastic fibers. The control groups showed a similar pattern of arrangement and thickness of the elastic fibers. The LDL KO groups showed more spaced fibers and thicker vessel walls compared to the controls. Photomicrographs, H&E. Calibration Bar = 100 µm. Sedentary control (S-C), ovariectomized sedentary control (OS-C), ovariectomized trained control (OT-C), sedentary LDL KO (S-LDL KO), ovariectomized sedentary LDL KO (OS-LDL KO) and ovariectomized trained LDL KO (OT- LDL KO).

The histopathological analysis showed that the animals from the control groups (S-C, OS-C and OT-C) did not exhibit changes in the elastic fiber arrangement and thickness pattern. However, the dyslipidemic groups (S-LDL KO, OS-LDL KO and OT-LDL KO), showed greater spacing between elastic fibers (Figure 1).

### Thickness of the tunica media - intima (µm)

**Figure 2:**
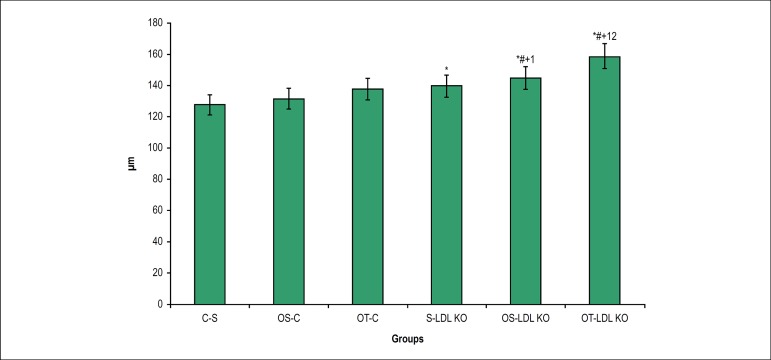
Thickness of the tunica media-intima (µm) in the studied groups. Values are expressed as M ± SD *p < 0.05 vs. S-C; #p < 0.05 vs. OS- C; +p < 0.05 vs. OT-C; ¹p < 0.05 vs S-LDL KO; 2p < 0.05 vs OS-LDL KO.

We observed a significant increase in the thickness of the tunica media and intima in dyslipidemic animals, when compared to the control group animals. The ovariectomy and the exercise in the LDL KO groups were a determining factor for the increase of this variable. In both control and LDL KO groups the training exercise did not reverse this process (Figures 1 and 2).

### Volume density of types I and III collagen fibers in the intima-media and adventitia tunica

**Figure 3:**
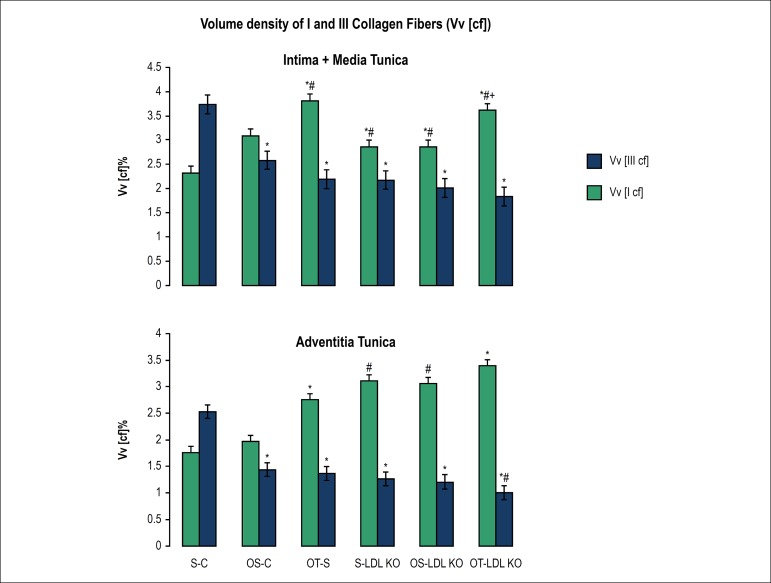
Volume density of types I and III collagen fibers (Vv[cf]) in Intima-Media and Adventitia Tunica of ascendant aorta. Values are expressed as M ± SD. *p < 0.05 vs. S-C; #p < 0.05 vs. OS-C; +p < 0.05 vs. OT-S.

Similar behavior of the type III collagen fiber was observed between the intima-media and adventitia tunica. We observed a significant decrease in the volume density of the type III collagen fiber in the LDL KO groups, when compared to the S-C (Figure 3).

The type I collagen fibers of the tunica adventitia and media-intima showed increase in volume density, influenced by training in the Control groups. The dyslipidemia induces an increase in type I collagen fibers in the LDL KO groups when compared to the Control groups and did not undergo any change by ovariectomy, or by training (Figure 3).

### Immunohistochemical analysis

**Figure 4:**
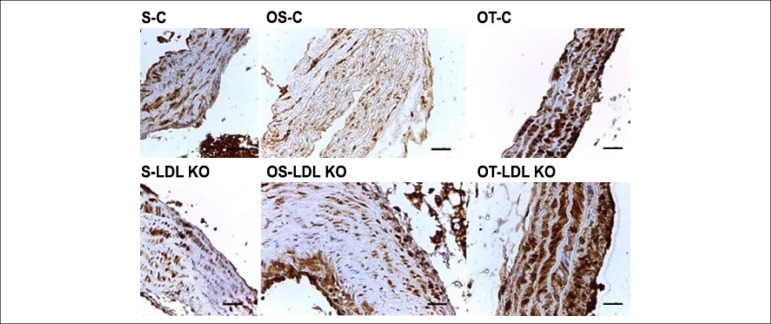
Photomicrographs of cross-sections of the aorta of mice submitted to immunohistochemical reaction for 8-OHdG. The immunoexpression of 8-OHdG was observed in all investigated groups. The staining was moderate for LDL KO and Control groups, both the S and the OS. However, for the O and T groups, the observed staining was intense. Calibration bar = 100 µm.

Figure 4 shows the tissue staining caused by the oxidative stress marker 8-OHdG. Note that the immunoexpression of the marker occurred in all groups. The staining was moderate for LDL KO and Control groups, both the Sedentary and the Ovariectomized Sedentary groups. However, for the Ovariectomized and trained groups, the observed staining was intense.

**Figure 5:**
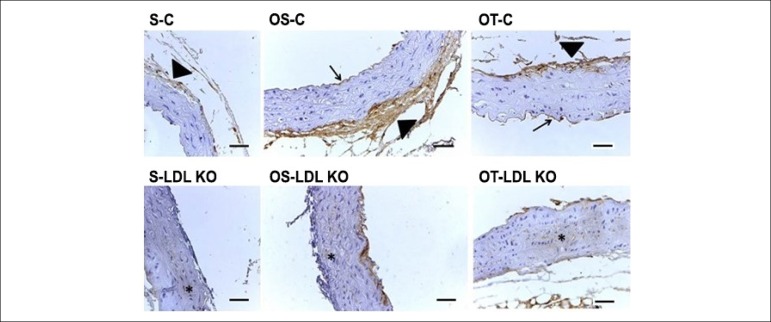
Photomicrographs of cross-sections of the ascending aorta of mice submitted to immunohistochemical reaction for MMP-2. Note the presence of MMP-2 immunoexpression in the tunica intima (arrow) and adventitia (arrowhead) in all control groups (S-C, OS-C and OT-C). In general, the MMP-2 immunoexpression in the LDL KO groups was observed in the intima and adventitia layers, as well as in the middle layer of the aorta (*), which did not occur in any animal of the control groups. Calibration bar: 100 µm.

**Figure 6:**
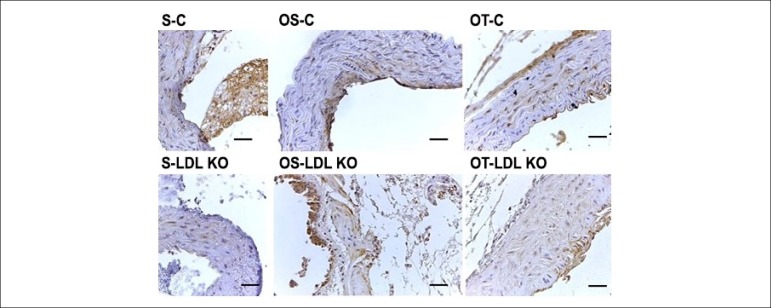
Photomicrographs of cross-sections of the ascending aorta of mice submitted to immunohistochemical reaction for MMP-9. Note the presence of immunoexpression in all layers, heterogeneously, in all groups. Calibration bar: 100 µm.

In all control groups (S-C, OS-C and OT-C) the immunoexpression of MMP-2 occurred both in the tunica intima (arrow) and in the tunica adventitia (arrowhead) of the ascending aorta. The LDL groups, in general, showed MMP-2 immunoexpression beyond the tunica intima and adventitia, but also in the tunica media of the aorta (although very slightly), which was not observed in any groups of control animals (Figure 5). The MMP-9 was expressed in all layers of the ascending aorta of all groups; however, the distribution was heterogeneous (Figure 6).

**Figure 7:**
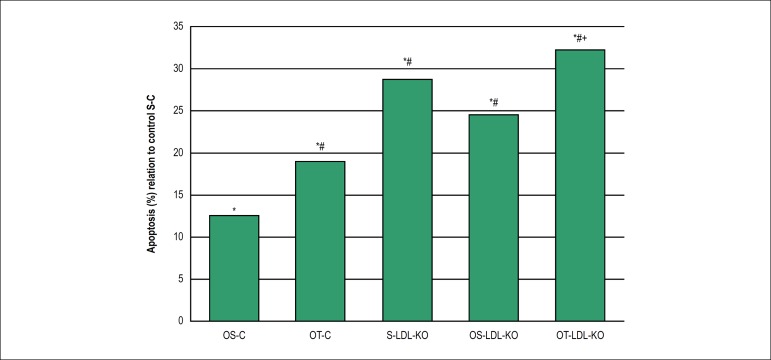
Relative frequency (%) of apoptotic cells in the ascending aorta of the control sedentary group (S-C) vs. the different evaluated groups as evidenced by the TUNEL assay. *p < 0,05 vs. S-C, #p < 0,05 vs. OS-C, +p < 0,05 vs. OS-LDL-KO. Values are expressed as mean ± SD (p < 0,05).

Apoptotic cells were distributed in the media-intima layer of the ascending aorta in all studied groups. The comparation of relative frequency showed that Ovariectomy statistically increased apoptosis in +12,6% in the sedentary group (OS-C) and +19% in physical training group (OT-C) when compared to the apoptosis of the sedentary control group (S-C) (Figure 7). Among the knockout groups, apoptosis rates were higher than the respective controls, regardless if the mice were ovariectomized or not. Thus, the knockout sedentary group increased apoptosis frequency in +28,8% when compared with the control group. Ovariectomy and knockout statistically increased the apoptosis in +24,5% in the sedentary (OS-LDL KO) and +32,3% in the physical training (OT-LDL KO) groups, when compared to the sedentary control group (S-C). No significant change was observed when comparing the knockout sedentary group (S-LDL-KO) to knockout ovariectomy sedentary (OS-LDL KO) or physical training (OT-LDL KO) groups, with decreased apoptosis in -4,3% and improved apoptosis in +3,5%, respectively. However, the relative frequency of ovariectomy, sedentary and physical training knockout groups showed to be statistically increased in +7,8% (Figure 7).

## Discussion

The main objective of this study was to verify the effects of moderate aerobic training on the ascending aorta of ovariectomized female mice, knockout for the low-density lipoprotein receptor LDL through the analyses of types I and III collagen fibers, the expression of 8-OHdG oxidative stress markers of MMP-2 and MMP-9 metalloproteinases.

Collagen and elastin are major structural and functional components of the arterial wall. These components actively participate in arterial wall remodeling in response to hemodynamic alterations and during atherogenesis.

The histopathological analysis did not show any morphological changes in the control group. However, the LDL KO group showed increased thickness and larger spacing between the elastic fibers. Some studies have demonstrated an association between an increase in intima-media thickness and the occurrence of cardiac events,^28^ which confirmed the existence of the association between increased intima-media thickness of the carotid and the presence of cardiovascular risk factors, including infection and inflammation markers. Collagen I is mostly a structural collagen; collagen III, in turn, is more frequent in pathological processes. Our results have shown that the number of cells in apoptosis were significantly lower in the control group and remained constant in the Control groups (OS-C and OT-C) and the LDL knockout groups (S-LDL KO, OS-LDL KO and OT-LDL KO), without significant differences in the percentage of apoptotic cells between the Control or LDL Knockout groups for each parameter used. However, the apoptosis process was greater in animals of LDL knockout groups, when compared with animals of the control groups, regardless if they were ovariectomized or not. This suggests that ovariectomy was not a major factor in the processes of apoptosis induction in the aorta.

The high levels of LDL in the bloodstream may have been the apoptosis-inducing factor in the endothelium and the tunica media of the ascending aorta in LDL knockout groups. The endothelial dysfunction induced by LDL oxidation (ox-LDL) has been associated to the pathogenesis of atherosclerosis and other vascular disorders. It is known that the ox-LDL activates ROS release and has been associated to apoptosis and endothelium damage.^29^ The apoptosis of vascular smooth muscle cells (VSMC) is associated with the occurrence of vascular diseases. In atherosclerosis, cell apoptosis induction has been associated with atherosclerotic plaque rupture, clotting, vessel remodeling, tunica media atrophy, aneurysm formation and calcification.^30^ Furthermore, in various human diseases such as Marfan syndrome and cystic necrosis of the tunica media (CMN), the apoptotic processes results in higher breakage of the elastic fibers, abnormal extracellular matrix deposition and tunica media expansion.^31^ In this environment, the release of interleukin IL1α and IL8, as well as the chemoattractant protein expression of monocytes (MCP-1) occurs during the VSMC apoptosis, which have causes infiltrating macrophages *in vivo*, increasing the observed tissue damage.^32^

The animals of the OT-C group showed a higher number of apoptotic cells compared to the OS-C and S-C groups (p < 0,05). Physical activity has been associated with increased apoptosis levels in rats’ thymocyte, mice skeletal muscle and lymphocytes.^33,34^ Oxidative stress resulting from metabolism in physical activities has been largely associated to apoptosis. In patients with cardiovascular diseases, the deficiency in nitric oxide (NO) production, associated with oxidative stress, results in a decline of NO bioavailability, inducing apoptosis of endothelial cells and therefore, resulting in endothelial dysfunction.^35^ The ovariectomized animals showed a higher percentage of apoptotic cells than the trained and sedentary control groups (OT-C and OS-C). This suggests that decreased hormone production may be related to a reduction in antioxidative effects on the body.

8-OHdG is one of the main oxidative products of DNA, which is considered a reliable marker of oxidative DNA damage. Thus, 8-OHdG has been widely used as a sensitive biomarker of oxidative stress.^36^ The immunohistochemical analysis of 8-OHdG showed staining in all groups; however, the trained groups showed higher intensity staining. Goto et al.,^9^ found that high-intensity exercise increases 8-OHdG levels in the plasma, which explains the higher degree of staining in the trained groups.

There were differences in MMP-2 expression, and the control group showed staining in the intima and adventitia layers, while the LDL KO group showed staining in the tunica media. According to Sakalihasan et al.,^37^ this occurs because the atherosclerotic lesion causes the migration of MMP-2 at the ends and at a lower quantity in normal tissue, for the tunica media, i.e., the formation of atherosclerotic plaques activates a set of chain reactions, which can increase the amount of MMP-2 present in the tissue. With regard to the MMP-9 expression, all groups showed tissue staining, but without a pattern. No evidence explaining this heterogeneous staining was found.

## Conclusion

The experimental model analyzed shows histomorphometric changes with increased expression of 8-OHdG in trained groups. An increase in the apoptosis rate was observed in the trained groups and the LDL KO ovariectomized group. The groups stained with MMP-2 showed migration and its increased expression in the tunica media of LDL KO groups. However, the MMP-9 staining appeared in all groups, but did not follow a homogeneous pattern. Finally, studies on the expression of metalloproteinases in cardiac muscle tissues with atherosclerosis are very scarce, suggesting the need for further studies to investigate the issue. Thus, the results described herein suggest that moderate-intensity aerobic exercise in ovariectomized mice associated to an increase in LDL rates possibly increases oxidative stress and apoptosis induction. 

The evaluation of the parameters under study (MMPs, apoptosis and 8-OHdG) was performed by immunohistochemistry. However, other more sensitive technologies (such as molecular biology ones) could be used in these assessments, leading to more precise results and interpretations.
